# Livestock guardian dogs as surrogate top predators? How Maremma sheepdogs affect a wildlife community

**DOI:** 10.1002/ece3.2412

**Published:** 2016-08-30

**Authors:** Linda van Bommel, Chris N. Johnson

**Affiliations:** ^1^ School of Biological SciencesUniversity of TasmaniaHobartTas.Australia; ^2^ Fenner School of Environment and SocietyAustralian National UniversityCanberraACTAustralia

**Keywords:** detection probability, large herbivore, LGD, LPD, mesopredator, top predator, trophic cascade

## Abstract

Use of livestock guardian dogs (LGDs) to reduce predation on livestock is increasing. However, how these dogs influence the activity of wildlife, including predators, is not well understood. We used pellet counts and remote cameras to investigate the effects of free ranging LGDs on four large herbivores (eastern gray kangaroo, common wombat, swamp wallaby, and sambar deer) and one mesopredator (red fox) in Victoria, Australia. Generalized mixed models and one‐ and two‐species detection models were used to assess the influence of the presence of LGDs on detection of the other species. We found avoidance of LGDs in four species. Swamp wallabies and sambar deer were excluded from areas occupied by LGDs; gray kangaroos showed strong spatial and temporal avoidance of LGD areas; foxes showed moderately strong spatial and temporal avoidance of LGD areas. The effect of LGDs on wombats was unclear. Avoidance of areas with LGDs by large herbivores can benefit livestock production by reducing competition for pasture and disease transmission from wildlife to livestock, and providing managers with better control over grazing pressure. Suppression of mesopredators could benefit the small prey of those species. *Synthesis and applications:* In pastoral areas, LGDs can function as a surrogate top‐order predator, controlling the local distribution and affecting behavior of large herbivores and mesopredators. LGDs may provide similar ecological functions to those that in many areas have been lost with the extirpation of native large carnivores.

## 1 Introduction

Livestock guardian dogs (LGDs, *Canis familiaris*) have been used for centuries to protect livestock from predators and thieves in Europe and Asia, and their use is increasing in other parts of the world (Gehring, Vercauteren, & Landry, 2010; Rigg, 2001). Experimental and comparative evidence shows that these dogs can be effective in protecting a range of livestock species from several types of predators, both on small properties and extensive livestock operations (van Bommel & Johnson, 2012; Gehring et al., 2010; Rigg, 2001). This implies that LGDs influence the movements and behavior of predators in some way. However, it is currently unknown how, or to what extent, LGDs affect predator behavior. In addition, few studies have investigated the influence of LGDs on the movements and behavior of other species of wildlife that coexist with livestock.

Livestock guardian dogs could affect wildlife in several ways. Most LGDs do not need to hunt for food, as they are regularly fed by their owners (Van Bommel, 2010), but predation can still occur: LGDs have been reported killing a range of predator and prey species, including deer fawns (*Odocoileus hemionus*), marmots (*Marmotta* spp), coyotes (*Canis Latrans*), and black‐backed jackals (*Canis mesomelas*) (Black & Green, 1985; Hansen & Smith, 1999; Potgieter, Kerley, & Marker, 2015; Timm & Schmidtz, 1989). LGDs could also chase or otherwise harass wildlife. Harassment of wildlife, including predators, by LGDs is evidently common (e.g., Coppinger, Coppinger, Langeloh, Gettler, & Lorenz, 1988; Gingold, Yom Tov, Kronfeld Schor, & Geffen, 2009; Hansen & Smith, 1999). Harassment is most likely to be directed at medium to large sized animals that are easily detected, or predators that might be perceived as threatening to livestock. To reduce harassment or risk of attack other species might change their behavior, decreasing their activity in areas used by LGDs, or avoiding these areas either spatially or temporally. Such responses might be especially strong for species with an evolutionary background as prey or competitors of large canids.

Most previous studies of interactions between LGDs and wild herbivores have found strong effects, but these studies have investigated situations where the LGDs are confined to small areas with their livestock: 1.2 ha and 10–40 ha in two studies of white‐tailed deer (*Odocoileus virginianus*), and a maximum of 240 ha for mountain gazelle (*Gazella gazella*; Gehring, Vercauteren, Provost, & Cellar, 2010; Gingold et al., 2009; Vercauteren, Lavelle, & Phillips, 2008). One study found an effect of free ranging LGDs on red deer (*Cervus elaphus*) and roe deer (*Capreolus capreolus*) on a larger scale (Dorresteijn et al., 2015).

In our study, we investigated LGDs that ranged freely over large areas, readily crossing stock fences that limited movements of their livestock. A diverse community of wildlife used the areas on and around our research properties, and large tracts of natural vegetation remained in the area. Movement of wildlife was unrestricted. This enabled us to study the influence of LGDs at scales relevant to the typical ranging behavior and habitat utilization patterns of large mammalian wildlife.

**Figure 1:**
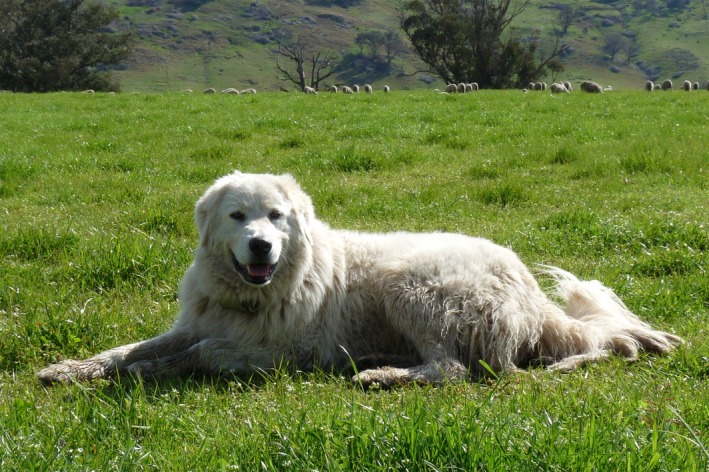
Maremma sheepdog looking after his flock on Heatherlie

Five species of wildlife were included in the study: one introduced mesopredator, the red fox (*Vulpus vulpus*); three native large herbivores, the eastern gray kangaroo (*Macropus giganteus*), common wombat (*Vombatus ursinus*), and swamp wallaby (*Wallabia bicolor*); and one introduced large herbivore, the sambar deer (*Rusa unicolor*). We used pellet counts and remote cameras to determine how the presence of LGDs (Maremma sheepdogs, “Maremmas,” Fig. 1) affected the activity of these species in time and space. We wanted to know whether these species showed avoidance of the areas used by Maremmas, whether they were excluded from such areas altogether, or whether they were unaffected by the presence of Maremmas. If they showed avoidance, we wanted to investigate whether this avoidance was spatial, temporal or both.

## 2 Methods

### 2.1 Research properties and distribution of guardian dogs

**Figure 2:**
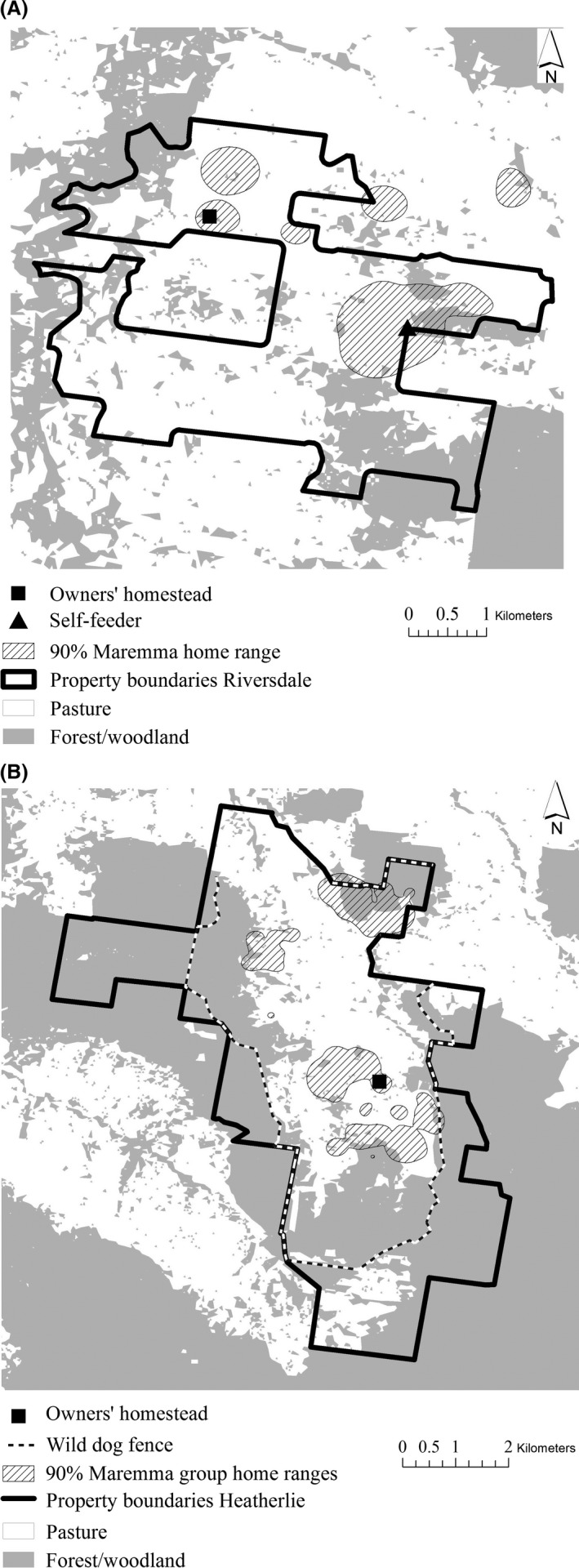
The two research properties (A) Riversdale and (B) Heatherlie

We worked on two properties in northeast Victoria, Australia (Fig. 2). Riversdale covered 1,214 ha, of which a 728 ha area was predominantly used to graze 1500 merino sheep (*Ovies aries*). Four Maremma sheepdogs guarded all sheep on the property and also visited sheep on two neighboring properties. Heatherlie covered 2,428 ha and ran 6,000–8,000 merino sheep, along with seven Maremmas. On Riversdale, all Maremmas functioned as one social group. On Heatherlie the Maremmas initially formed three distinct social units operating at opposite ends of the property 1–3 km apart. After 3 months, two groups merged, leaving two groups operating 3 km apart. On both properties, the Maremmas were free ranging, readily crossing stock fences but generally concentrating their activity in areas used by their sheep. The properties were approximately 15 km apart and had similar environmental features. They were hilly, with elevation between ~200 and 900 masl. Large tracts of uncleared native vegetation remained on both properties and in the surrounding area, and they were surrounded by a mixture of other grazing properties, natural vegetation, and pine plantations. For more detailed information on the research properties and the management of the Maremmas, see van Bommel and Johnson (2014).

The main predators of livestock in the area were wild dogs (*C. familiaris* including dingoes *Canis dingo* and hybrids), which had caused large losses of sheep on both properties before introduction of the Maremmas (in 2006 on Riversdale and 2009 on Heatherlie). Smaller predators especially red foxes, cats (*Felis catus*), and wedge‐tailed eagles (*Aquila audax*) were also present. Trapping, shooting, and baiting of wild dogs still occurred in the areas around the properties. Parts of Heatherlie were bounded by an electrified wild‐dog exclusion fence. The main wild large herbivores in the area were eastern gray kangaroos, swamp wallabies, common wombats, and sambar deer.

All Maremmas on Riversdale, and five Maremmas on Heatherlie, were fitted with GPS tracking collars (Telemetry Solutions, Concord, CA, USA) for a minimum of 4 weeks before the start of the wildlife surveys. The collars were set to take a location every 30 min, 24 hr a day. These data were used to calculate a fixed kernel home range (Worton, 1989) for each dog individually and for each group by pooling the tracking data of all members. See van Bommel and Johnson (2014) for details on methods used to calculate home ranges.

### 2.2 Data collection

#### 2.2.1 Pellet counts

Pellet counts were done in Maremma ranges (within the 95% kernel isopleths area of the group and in ecologically similar areas without Maremmas (outside of the 100% kernel isopleth area of the group), to obtain an index of activity of wildlife. Both inside and outside Maremma ranges, areas were selected that experienced the least disturbance from humans on a day to day basis. The counts were made in four locations: one on Riversdale, and three on Heatherlie (one for each separate group of Maremmas). Pellet accumulation was measured along 100 m transects with a width of 2 m. There were 10 transects for each area with or without Maremmas in each of the four locations. The start and end positions of each transect were marked and entered in a GPS. Each transect was cleared of all pellets, and left undisturbed for 7 days before pellets were counted. Pellets were identified to species by their size, shape, texture, color, and smell, following Trigg (2005). Wombat pellets were identified by their cube shape, green – brown uniform plant material present inside the scat and, especially for non‐cube shaped pellets, the unique wombat smell. Eastern gray kangaroo scats were identified by their oval, round, or square shapes, and the green – brown uniform plant material present in the scat. Swamp wallaby pellets were distinguished from kangaroo pellets by the brown, course, and less uniform texture inside the scats. Deer were probably not present in the areas of the transects, and due to the similarity of their pellets to sheep pellets, they were not included in the pellet counts. Counts were of the total number of individual pellets for each species. Counts were made in July 2011 on Heatherlie and in February 2012 on Riversdale.

At the start, end, and center of each transect, the following variables were recorded in a square meter quadrat: percentage of ground cover (all vegetation), percentage of grass cover, and height of the ground vegetation (class 1: <10 cm; class 2: >10 cm). Values from the three quadrats were averaged for each transect. Transect locations were entered in ArcGIS (ESRI, 2011), and the distance to the nearest forest or woodland cover was measured from a satellite imagery layer of each property (SpotMaps, 2011). The Maremma group home ranges were divided into 10 kernel isopleth areas, one area for each 10% increase in probability of occurrence; for each transect, we determined the kernel isopleth. All pellet counts and collection of additional variables were made by one person (LvB).

#### 2.2.2 Camera traps

Covert II (DLC Trading Co, Lewisburg, KY, USA) motion‐triggered cameras were set up in areas containing Maremmas, and in matched areas without Maremmas, defined as for the pellet counts. A total of 25 cameras were deployed simultaneously, first on Riversdale, and then on Heatherlie (Table 1).

**Table 1:** Details of the camera survey

	Riversdale	Heatherlie
No. cameras in Maremma home range	12	4 each for three Maremma groups
No. cameras outside Maremma home range	13	4 for two Maremma groups, 5 for one group
Deployment period	August 2012–December 2012	June 2011–February 2012
Average no. cameras operational	17; 5 inside and 12 outside of Maremma home range	19; 8 inside and 9 outside of Maremma home range^a^
Total number of trap nights	2,386	4,491

a Camera failures were evenly distributed over the three groups.

Locations in each area (with or without Maremmas) were chosen to give an even distribution of cameras. Cameras were checked every 6 weeks, at which time all reasonable care was taken to leave as little human scent at the camera site as possible. Cameras were set to take a 10 s video when triggered, with a minimum of 1 min delay between consecutive movies to reduce repeat triggers by the same individual. No lures were used during the survey. For each camera survey site, the general vegetation type was noted (pasture/woodland/forest).

### 2.3 Data analysis

#### 2.3.1 Pellet counts

To determine whether the sampled areas inside the Maremma home range were different from the areas outside of these, two‐way ANOVA was used to test for statistical difference in percentage ground cover, percentage grass cover and distance to cover, accounting for dog group. Pearson's chi‐square was used to test this for vegetation height.

Only pellet counts from kangaroos and wombats could be analyzed, as sample sizes from other species were too small. For each species, total number of pellets per transect was entered in a generalized mixed model as the dependent variable. Two covariates were considered *a priori* to potentially influence the pellet counts: “location in the Maremma group home range” (1 if outside Maremma areas, otherwise a kernel isopleth value), and “sheep present” (binary variable). Due to the low sample size in this study (4 dog groups), only single explanatory variables were added for each species in a model, in order to minimize the number of estimated parameters. Therefore, for each species, three models were created: one with each of the covariates, and one without explanatory variables. To account for variability between dog groups and properties/years, the variable [location] was nested in the variable [dog group], and entered as a random variable in all models. These models represent the hypotheses in which (1) the location in the Maremma group home range is the most important determinant of finding pellets; (2) the presence of sheep is the most important determinant of finding pellets; (3) neither covariate is important. The models were ranked according to their Akaike information criterion (AIC) value (Burnham & Anderson, 2002). Analysis was conducted in R (R Core Team, 2013), using the package “lme4” (Bates, Maechler, Bolker, & Walker, 2015).

#### 2.3.2 Camera traps

The time, date, and species were recorded for each video. Fourteen Mammal species and a variety of birds were detected (Table 2). Analyses were restricted to four species of wild large herbivores (eastern gray kangaroo, common wombat, swamp wallaby, and sambar deer), and one small carnivore (red fox). Wild dogs and feral cats were not detected in sufficient numbers for analysis.

**Table 2:** The species detected on movement triggered cameras, and the number of detections. Number “a” represents the number used for one‐species occupancy models, “b” represents the number used for two‐species occupancy models

	Total	Riversdale	Heatherlie
Livestock (sheep and cattle)	7,150	1,142	6,012
Maremma *Canis familiaris*	109 (b:162)	21 (b:6)	88 (b:154)
Eastern gray kangaroo *Macropus giganteus*	461 (a:205, b:70)	94 (a:61, b:45)	367 (a:144, b:25)
Common wombat *Vombatus ursinus*	336 (a:216, b:122)	216 (a:137, b: 96)	120 (a:79, b:26)
Red fox *Vulpus vulpus*	123 (a:73, b:54)	44 (a:33, b:22)	79 (a:40, b:32)
Swamp wallaby *Wallabia bicolor*	108 (a:72)	82 (a:63)	26 (a:9)
Sambar deer *Rusa unicolor*	52 (a:44)	52 (a:44)	0 (a:0)
European rabbit *Oryctolagus cuninculus*	83	30	53
Brush‐tailed possum *Trichosurus vulpecula*	41	17	24
Rat *Rattus* sp.	16	0	16
Echidna *Tachyglossus aculeatus*	6	6	0
Feral cat *Felis catus*	6	3	3
Wild dog *C. familiaris/Canis dingo*	5	3	2
Bat *Chiroptera* sp.	1	1	0
Birds (eagles, corvids, song birds, parrots, kookaburras, ducks)	128	39	89

We analyzed data using one‐species single‐season occupancy models and two‐species single‐season occupancy models (Mackenzie et al., 2006). For each species, an occurrence matrix was created, recording detection or nondetection in each 24‐hr survey period (from 17:00 to 17:00 the following day). All models were constructed in PRESENCE 6.1 (Hines, 2006). We limited our analyses to probability of detection only, as the cameras were not sufficiently far apart to ensure spatial independence, as required for estimation of occupancy (Mackenzie et al., 2006).

#### 2.3.3 One‐species analysis

We used one‐species single‐season occupancy models to determine whether the location in Maremma home range had any influence on probability of detection of the species of wildlife in this study (Mackenzie et al., 2006). The same measure of location in Maremma home range was used as for the pellet counts. The model containing this covariate and a “base model” that estimated the probability of detection without any covariates, were ranked according to their AIC value (Burnham & Anderson, 2002). The camera survey effort was standardized to 133 days, representing the time at which the cameras had operated on Riversdale.

#### 2.3.4 Two‐species analysis

Two‐species single‐season analysis models the direct effect of the presence of one species on the probability of occurrence and detection of the other (Mackenzie, Bailey, & Nichols, 2004; Mackenzie et al., 2006). We used it to investigate the effect of the presence of Maremmas on the probability of detection of foxes, kangaroos and wombats. Wallabies and deer could not be analyzed, due to the absence of any detection within Maremma home ranges. Maremmas were detected on camera in low numbers. Therefore, we identified all instances in which a GPS collar from a Maremma had logged a location within 50 m of a camera, with the associated time and date, and added these to the detection data from the cameras. The analysis was therefore restricted to the time in which Maremmas were fitted with GPS collars during the time the cameras were operational, and standardized to a period of 3 months (88 days) on each property.

In PRESENCE, three alternative parameterizations are available for two‐species modeling; we used the simplest (Mackenzie et al., 2006). Our analyses were limited to probability of detection, therefore PsiA and PsiB were modeled independently and Phi was not estimated (Lazenby & Dickman, 2013; Mackenzie et al., 2006). The parameters that were estimated are: pA, the probability of detecting species A, given that species B is not present; pB, the probability of detecting species B, given that species A is not present; rA, the probability of detecting species A, given that both species are present; rB, the probability of detecting species B, given that both species are present; delta, species co‐detection, which is an expression of whether two species are detected independently at the survey sites. Values <1 indicate that a camera is less likely to detect a species during a 24 hr survey period if the other species was detected in that period (suggesting temporal avoidance or exclusion). Values >1 indicate that a camera is more likely to detect a species during a 24 hr survey period if the other species was detected in that period (suggesting temporal attraction).

Five covariates were considered a priori to potentially influence the probability of detection of all species at camera sites, in addition to the presence of the Maremmas. These were (1) study site/year; (2) dog group; (3) vegetation type (pasture, woodland, or forest); (4) livestock type (sheep, cattle, or no livestock); (5) time since the camera was last checked (days). Due to the low sample size in this study, only single covariates were added for each species in each model, in order to minimize the number of estimated parameters. For each species of wildlife, the models containing covariates, and a model without covariates were ranked according to their AIC value (Burnham & Anderson, 2002). To further explore the effect of Maremmas on the wildlife species of interest, three additional constraints were placed on each of the models that fell within 2 ∆AIC of the top model: (1) pA = rA, (2) pB = rB, and (3) delta = 1 (Lazenby & Dickman, 2013). These models were ranked with the unrestrained models according to AIC value.

## 3 Results

### 3.1 Pellet counts

No significant habitat differences were found between the transects inside and outside of the Maremmas’ range, for percentage ground cover (*F*_(1,75)_ = 0.20, *p* > .05), percentage grass cover (*F*_(1,75)_ = 0.00, *p* > .05), distance to cover (*F*_(1,75)_ = 0.18, *p *> .05) or vegetation height (*χ* ^2^_(9, *n* = 80)_ = 6.28, *p* > .05).

For kangaroos, the model including the explanatory variable “location in Maremma home range” ranked the highest, with the nearest contender (containing the explanatory variable “sheep present”) 35.7 ΔAIC removed. For wombats, the model containing the explanatory variable “sheep present” ranked the highest, with the nearest contender (containing the explanatory variable “location in Maremma home range”) 74.7 ΔAIC removed. For both species, the likelihood of finding pellets increased with an increasing distance from the core of the Maremma home range and was higher outside of areas containing sheep.

### 3.2 Camera traps – one‐species analysis

**Figure 3:**
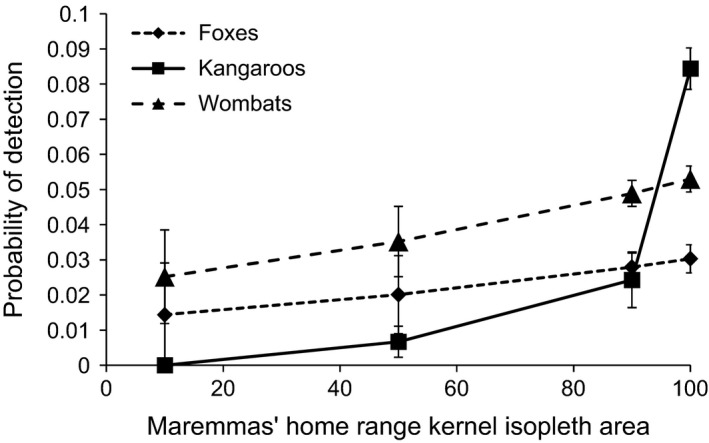
The probability of detecting foxes, kangaroos and wombats in relation to the location in the Maremmas’ home range, as represented by the kernel isopleth areas. The 10% location is the core of the Maremmas’ range, the 100% location is on the edge and outside the Maremmas’ range

Swamp wallabies and deer were never detected within Maremma ranges and could therefore not be modeled in this analysis. For foxes and kangaroos, the model containing the Maremma home range covariate had an AIC >25 units lower than the base model (Table 3), indicating the Maremma home range model is a better fit for the data. For both species, the probability of detection decreased toward the center of the Maremma home range (Fig. 3). For wombats, the probability of detection also decreased toward the center of the Maremma home range (Fig. 3). However, for wombats the model containing Maremma home range only ranked slightly higher than the base model (Table 3), indicating this covariate did not have a strong effect.

**Table 3:** The one‐species models, shown for each species

	Model covariates	AIC	Delta AIC	AIC weight	No. par
Foxes	m	710.78	0.00	1.00	3
	BM	738.75	27.97	0.00	2
Kangaroos	m	1,492.45	0.00	1.00	3
	BM	1,517.66	25.21	0.00	2
Wombats	m	1,708.62	0.00	0.53	3
	BM	1,708.89	0.27	0.47	2
Wallabies deer	Wallabies and deer were never detected within the Maremma home range and could therefore not be modeled				

“m” location in the Maremma home range; “BM” (base model) assumes a constant probability of detection for all cameras in the survey.

### 3.3 Camera traps – two‐species analysis

The top ranking two‐species models for Maremmas and foxes, Maremmas and kangaroos, and Maremmas and wombats are shown in Table 4. The outputs of the top ranking model are shown in Table 5. For Maremmas, livestock type was always an important covariate in the models; Maremmas were more likely to be detected with sheep.

**Table 4:** The highest ranking two‐species models (all models within 2 ∆AIC of the top model), and the nearest contender, modeling detection probabilities of Maremmas and foxes, Maremmas and kangaroos, and Maremmas and wombats

Models	AIC	Delta AIC	AIC weight	No. Par
Maremmas – Foxes				
pM(l), pF(vt), rM(l), rF(vt), delta()	1,478.96	0.00	0.68	10
*pM(l), pF(), rM(l), rF(), delta()*	*1,481.95*	*2.99*	*0.15*	*9*
Maremmas – Kangaroos				
pM(l), pK(l), rM(l), rK(l), delta()	1,470.23	0.00	1.00	11
*pM(l), pK(p), rM(l), rK(p), delta()*	*1,520.89*	*9.88*	*0.00*	*10*
Maremmas – Wombats				
pM(l), pW(p), rM(l), rW(p), delta()	1,840.24	0.00	0.51	10
pM(l), pW(p), rM(l), rW(p), delta=1()	1,840.31	0.07	0.49	10
*pM(vt), pW(p), rM(vt), rW(p), delta()*	*1,866.48*	*26.24*	*0.00*	*10*

M, Maremma; F, fox; K, kangaroo; W, Wombat. p, the detection probability of the species if the other species is not present; r, the detection probability of the species if both species occur at the site. The covariates included in the models are: vt, vegetation type; p, property; l, livestock type and d, number of days since the camera site was last checked. The models in italic values represent the nearest contender to the models that fall within 2 ΔAIC of the top model for each two‐species combination.

**Table 5:** The outputs of the top ranking models for the two‐species analysis of Maremmas and foxes, Maremmas and kangaroos, and Maremmas and wombats

Maremmas – Foxes				
*pM(l), pF(vt), rM(l), rF(vt), delta()*				
pM	pF	rM	rF	Delta
S 0.822 (0.090)	WL 0.009 (0.005)	S 0.126 (0.012)	WL 0.042 (0.009)	0.277
C 0.318 (0.097)	P 0.004 (0.003)	C 0.014 (0.010)	P 0.021 (0.005)	(0.270)
NS 0.055 (0.034)		NS 0.001 (0.002)		
Maremmas – Kangaroos				
*pM(l), pK(l), rM(l), rK(l), delta()*				
pM	pK	rM	rK	Delta
S 0.045 (0.009)	S 0.046 (0.013)	S 0.262 (0.020)	S 0.005 (0.002)	0.000
C 0.0007 (<0.001)	C 0.132 (0.036)	C 0.005 (0.003)	C 0.016 (0.006)	(<0.001)
NS 0.0009 (<0.001)	NS 0.254 (0.043)	NS 0.006 (0.003)	NS 0.036 (0.008)	
Maremmas – Wombats				
*pM(p,l), pW(p,vt,l), rM(p,l), rW(p,vt,l), delta=1()*				
pM	pW	rM	rW	Delta
S 0.400 (0.040)	R 0.036 (0.008)	S 0.097 (0.012)	R 0.166 (0.019)	0.870 (0.461)
C 0.016 (0.012)	H 0.005 (0.002)	C 0.003 (0.002)	H 0.026 (0.005)	
NS 0.078 (0.043)		NS 0.013 (0.007)		

p, probability of detecting the species if the second species is not present; r, the probability of detecting the species if both species occur. Delta is a measure for species co‐detection. M, Maremma; F, fox; K, kangaroo; W, wombat. l, livestock type (S – sheep, C – cattle, NS – no livestock), vt, vegetation type (WL – woodland, P – open pasture, F – forest). p; property (R – Riversdale, H – Heatherlie). Numbers in brackets represent standard errors.

#### 3.3.1 Maremmas – Foxes

Foxes were detected more often at camera sites if Maremmas had been detected, but Maremmas were detected less often at cameras if foxes had been detected. The probability of detecting both Maremmas and foxes at the same site within a 24‐hr period was less than the probability of detecting either species alone; delta was 0.28. Vegetation type was the most important covariate for foxes; during the period of analysis foxes were never detected in forest, and their probability of detection was higher in woodland than in open pasture.

#### 3.3.2 Maremmas – Kangaroos

**Figure 4:**
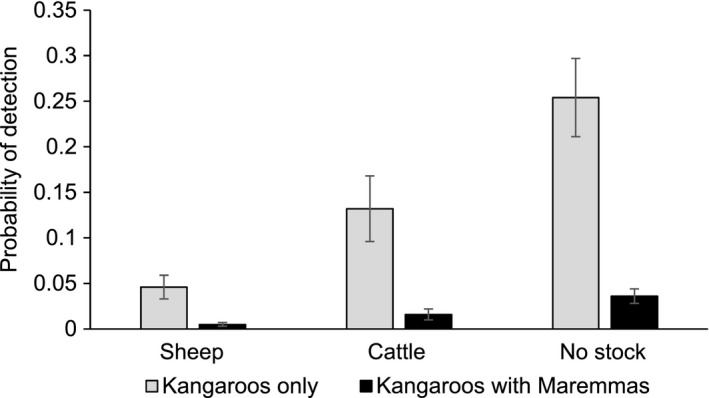
The probability of detecting kangaroos in areas where Maremmas have been detected and in areas where Maremmas have not been detected

Kangaroos were detected consistently less often at camera sites where Maremmas had been detected (Fig. 4). The probability of detecting kangaroos at sites were Maremmas had been detected was often less than 20% of that at sites where they had not been detected. Maremmas were consistently detected more often at camera sites where kangaroos had been detected. Kangaroos and Maremmas were never detected at the same site within a 24‐hr period; delta was 0. Livestock type was an important covariate for kangaroos; their probability of detection was lowest in sheep areas and highest in areas without livestock.

#### 3.3.3 Maremmas – wombats

Wombats were detected consistently more often at camera sites where Maremmas had been detected, but Maremmas were detected less often at sites where wombats had been detected. The chance of detecting wombats and Maremmas at the same site within a 24 hr period was equal to the chance of detecting each species alone; delta was high close to 1 in the top ranking model, and the same model with the constraint of delta = 1 ranked very close to the top ranking model. Study site was an important covariate for wombats; their probability of detection was lower on Heatherlie.

## 4 Discussion

Most wildlife species in this study showed avoidance of Maremmas’ ranges. This avoidance was strongest in wallabies and deer, which appeared to be completely absent from the areas used by Maremmas. Kangaroos, wombats, and foxes were not fully excluded from Maremma areas, but spatial and temporal avoidance of Maremmas was found for kangaroos and foxes. Little effect of Maremma presence was found for wombats.

The strong effect of Maremmas on the distribution of wallabies and deer in this study was probably facilitated by the habitat preference of these species. Wallabies and deer prefer forest or woodland habitats over open areas (Hill & Phinn, 1993; Kushwaha, Khan, Habib, Quadri, & Singh, 2004; Porwal, Roy, & Chellamuthu, 1996). Maremma ranges mostly focused on livestock locations (Van Bommel & Johnson, 2014), and as livestock were generally not grazed in forest, the dogs’ ranges included only small areas of forest. Wallabies and deer were absent from these areas, but large tracts of forest remained outside of LGDs ranges, offering ample alternative living space and probably facilitating avoidance of LGDs. For sambar deer, grassland can also be an important foraging habitat (Forsyth, McLeod, Scroggie, & White, 2009). In this study, deer presence on grassland was very low, although on neighboring properties without LGDs, deer were regularly observed grazing in livestock paddocks at dusk.

Kangaroos, foxes, and wombats all prefer habitats matching the livestock grazing areas: open areas close to cover for kangaroos and wombats and a mosaic of forest or woodland and pasture for foxes (Moore, Coulson, & Way, 2003; Pita, Mira, Moreira, Morgado, & Beja, 2009; Roger, Laffan, & Ramp, 2007). Such habitats comprised large parts of the Maremmas’ ranges. Maremmas most strongly influenced kangaroos, followed by foxes, with little apparent effect on wombats. Kangaroos preferred areas without sheep, which could enhance the effect of the Maremmas, or might be a result of the presence of Maremmas with stock. Similar effects on kangaroos were found during an opportunistic Maremma removal experiment during this study, in which the Maremma was removed from her area while the sheep remained. The probability of detecting kangaroos increased after the Maremma had been removed, as did the probability of detecting wombats (L. van Bommel, unpublished data). However, there was little effect on foxes (L. van Bommel, unpublished data). For foxes, the presence of newborn lambs during the period of the research was probably highly attractive as a potential food source (Saunders, Gentle, & Dickman, 2010). Maremmas were more likely to be detected with sheep, and as two‐species models cannot differentiate between different locations within Maremmas’ ranges, these models led to the result that foxes do not appear to be spatially impacted by the presence of LGDs. However, foxes seem to favor locations toward the edges rather than the cores of Maremma ranges, as found in the one‐species models.

These results indicate that LGDs, as well reducing predation on livestock, can provide other benefits for the management of livestock and their ranges. Large herbivores like kangaroos and deer can compete with livestock for pasture, reducing the amount of feed that is available for stock (Edwards, Croft, & Dawson, 1996). LGDs could be utilized to limit the access of wild herbivores to livestock growing areas, allowing producers to more effectively regulate total grazing pressure and plan for resting of pastures on their property. In Australia, some farmers already use LGDs for this purpose (Van Bommel, 2010). LGDs could also be a valuable tool in disease management, by reducing disease transmission between wild and domestic herbivores. Ungulates are potential carriers of a range of diseases that can affect livestock, such as Johne's disease and bovine tuberculosis; transmission from wild to domestic herbivores would have a significant impact on the livestock industry (Kennedy & Allworth, 2000; Mackintosh, de Lisle, Collins, & Griffin, 2004). Vercauteren et al. (2008) and Gehring et al. (2010) found that LGDs can segregate white‐tailed deer from cattle in the USA, and thereby potentially facilitate disease control. Dorresteijn et al. (2015) found that the presence of LGDs had a negative effect on the occurrence of red deer and roe deer in forests in Romania. This is similar to the finding in our study, where the Maremmas excluded sambar deer from their ranges. This indicates that LGDs could play an important role in disease management. Negative impacts of LGDs on large herbivores could be undesirable if they conflict with conservation of those species (Gingold et al., 2009). However, proper training and management of LGDs to familiarize them with endangered or threatened species might make it possible to avoid negative impacts on such species.

Spatial and temporal avoidance by foxes of Maremmas gives some insight into how LGDs prevent livestock predation by smaller predators. Avoidance is probably an important part of this process. Gehring et al. (2010) also found that presence of LGDs reduced detection of mesopredators (foxes, raccoons, skunks). However, Dorresteijn et al. (2015) reported that the presence of LGDs increased the occurrence of foxes. This could be related to the habitat preference of foxes matching landscapes where there was a higher chance of LGDs occurring due to their use by livestock (Dorresteijn et al., 2015), as we also found in this study. In our study foxes were still present at the edge of the LGDs’ ranges, but livestock predation did not increase in those areas. Other factors must therefore play a role as well. The presence of LGDs could cause changes in fox behavior that this study could not detect; for example, foxes might be more vigilant and cautious when they perceive themselves to be in a LGDs’ range, which could limit their hunting behavior. If LGDs do affect the behavior of mesopredators in this way, they could benefit biodiversity by providing indirect protection to species that are prey of mesopredators. Gehring et al. (2010) found that paddocks with LGDs contained more ground nesting birds that experienced lower rates of predation compared to control paddocks, possibly due to the impact of LGDs on mesopredators. In Australia, foxes and feral cats (*F. catus*) have had devastating impacts on wildlife and are thought to be responsible for the decline and extinction of many species of small mammals, birds, and reptiles (Johnson, 2006). LGDs could potentially create refuges for species threatened by fox and cat predation. This could greatly benefit conservation and biodiversity over large tracts of pastoral land in Australia. However, more research is needed on this topic.

From an ecological point of view, LGDs can be viewed as surrogate top predator in pastoral areas. Top predators, such as wolves and dingoes, can have large ecological impacts not only by predation, but by creating a “landscape of fear” which regulates the local distribution and behavior of large herbivores and mesopredators (Letnic, Koch, Gordon, Crowther, & Dickman, 2009; Ripple et al., 2014). For example, herbivores and mesopredators might avoid areas with high risk of encountering a large predator, or reduce the amount of time spent foraging in areas where they perceive risk (Brown & Kotler, 2004). Fear‐induced behavioral changes in large herbivores and mesopredators can lead to a reduction of their impacts on plant communities and small prey (Berger, Gese, & Berger, 2008; Fortin et al., 2005). However, in pastoral areas the presence of a natural top predator is usually unacceptable due to the threat posed to stock. LGDs could offer a livestock‐friendly alternative, which, by reducing damaging incursions by wild predators, could allow those predators to persist outside of livestock areas, thereby contributing to predator conservation (Marker & Boast, 2015).

## Funding Information

Australian Research Council and the Hermon Slade Foundation.

## Conflict of Interest

None declared.
